# A Homœopathic Bibliography of the United States

**Published:** 1891-06

**Authors:** 


### BOOK NOTICES.

A Homœopathic Bibliography of the United States, from the year 1825 to 1891, inclusive. Containing alphabetical lists of Homœopathic Books, Magazines, and Pamphlets; also condensed statements, data, and histories of the Homœopathic Societies, Colleges, Hospitals, Asylums, Homes, Dispensaries, Pharmacies, Publishers, Directories, Legislation,

Principal Books written against Homœopathy, and Homœopathic Libraries now or at any time existent in the United States. Compiled and arranged by Thomas L. Bradford, M. D., 1862 Frankford Avenue, Philadelphia, Pa.

The desirability of publishing this book will be seen at once by every one having any love for, or pride in, Homœopathy. It will not be published until a sufficient number of subscribers have been received. It will make a book of from 400 to 500 octavo pages, will be well printed on good paper and substantially bound, and the price will be $3.00.

We hope our readers will all subscribe for this book at once that its publication may not be delayed.

